# Imaging mass spectrometry to visualise increased acetylcholine in lungs of asthma model mice

**DOI:** 10.1007/s00216-020-02670-0

**Published:** 2020-05-04

**Authors:** Takeshi Matsuda, Yuzo Suzuki, Tomoyuki Fujisawa, Yasunori Suga, Nobuyuki Saito, Takafumi Suda, Ikuko Yao

**Affiliations:** 1 Department of Optical Imaging, Institute for Medical Photonics Research, Preeminent Medical Photonics Education & Research Center, Hamamatsu University School of Medicine, 1-20-1 Handayama, Higashi-ku, Hamamatsu, 431-3192 Japan; 2 Department of Pharmaceutical Sciences, College of Pharmaceutical Sciences, Ritsumeikan University, 1-1-1 Nojihigashi, Kusatsu, Shiga 525-8577 Japan; 3 Second Division, Department of Internal Medicine, Hamamatsu University School of Medicine, 1-20-1 Handayama, Higashi-ku, Hamamatsu, 431-3192 Japan; 4 Nippon Boehringer Ingelheim Co., Ltd., 2-1-1 Osaki, Shinagawa-ku, Tokyo, 141-6017 Japan; 5 International Mass Imaging Center, Hamamatsu University School of Medicine, 1-20-1 Handayama, Higashi-ku, Hamamatsu, 431-3192 Japan; 6 Department of Biomedical Chemistry, School of Science and Technology, Kwansei Gakuin University, 2-1 Gakuen, Sanda, Hyogo 669-1337 Japan

**Keywords:** Imaging mass spectrometry (IMS), Fourier transform ion cyclotron resonance mass spectrometry (FT-ICR-MS), Acetylcholine (ACh), Asthma model mouse, Lung tissue, Imaging

## Abstract

Acetylcholine (ACh) is a crucial neurotransmitter that is involved in airway constriction. In fact, excessive ACh binding to M3 muscarinic receptor leads to airflow obstruction via smooth muscle contraction. Previous studies have suggested cholinergic malfunction in the pathogenesis of asthma; however, the distribution and abundance of ACh in asthmatic lungs remain unclear because of the challenges of imaging ACh in lung tissue. In this study, we successfully detected and visualised ACh in mouse lung tissue by using Fourier transform ion cyclotron resonance mass spectrometry (FT-ICR-MS). Here, we applied the ACh imaging method to the two groups of house dust mite-sensitised asthma model mice harbouring different inflammatory levels. The imaging results showed that the lungs of mice had a relatively uniform ACh distribution with some areas of heterogeneity. The lungs of asthma model mice had significantly more ACh than control mice, and the ACh increase was potentiated with intense eosinophil infiltration without acetylcholinesterase deficits. These results indicate that ACh hypersecretion is mediated by an increased infiltration of eosinophils in asthma aggravation. This study provides the first evidence that secreted ACh is elevated with asthma severity in the lungs of asthma model animals by a direct ACh imaging technique with FT-ICR-MS.

**Electronic supplementary material:**

The online version of this article (10.1007/s00216-020-02670-0) contains supplementary material, which is available to authorized users.

## Introduction

Asthma is a common disease that is characterised by chronic inflammation and increased airway hyperresponsiveness (AHR) [1–3]. Airway constriction is triggered by the contractions of smooth muscles in the airways. Moreover, acetylcholine (ACh) secreted from the vagus nerve is one of the most significant molecules that trigger such contractions. Indeed, anti-cholinergic drugs that block the M3 muscarinic ACh receptors (mAChR) can relieve asthma attacks and have a bronchodilating effect in the clinical use [4–6]. Furthermore, in previous studies using asthma model animals, anti-cholinergic agents were shown to inhibit airway inflammation and remodelling [1, 5, 7–10]. Taken together, these results support a critical role of ACh in the aspects of asthma pathogenesis, including bronchoconstriction, mucus secretion, inflammation and remodelling. Despite interest in ACh as a key molecule of asthma pathophysiology, few studies have explored ACh secretion sites within the lungs of asthma model animals, even in a normal model animals. Although the relationship between ACh and asthma has been previously explored by using ACh injection or electrical stimulation of the vagus nerve [1, 11, 12], there have been no studies to date that directly observed ACh in the lung tissue of asthma model animals. Thus, little to no data are available on the effects of ACh distribution or quantity in lungs on asthma pathogenesis.

**Fig. 1:**
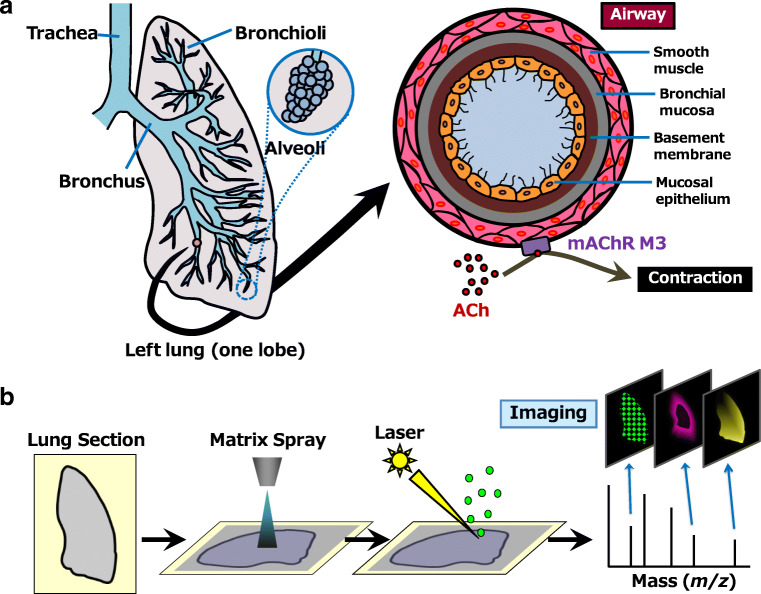
Schematic of mouse lung anatomy and imaging mass spectrometry. **a** Mouse lung anatomy. The rodent lung is composed of four right lobes and one left lobe. In this study, we used the left lobe as it is the largest. From centre to periphery, the lung tissue is segmentalised into trachea, bronchus, bronchioli and alveoli. The airway comprises four layers, namely, smooth muscle, bronchial mucosa, basement membrane and mucosal epithelium. The vagus nerve projects from the medulla to the lung and secretes acetylcholine (ACh) to the pulmonary parenchyma. The secreted ACh binds to M3 AChR of smooth muscles in the airway of the lung to regulate airway constriction. **b** Imaging mass spectrometry (IMS). This experimental technique enables simultaneous visualisation of the distribution and abundance of a target molecule in biological tissue

ACh is a crucial regulator of homeostatic autonomic functions in the mammalian respiratory system and induces respiratory contractions by binding to M3 mAChR on the smooth muscles of the bronchial pathway [5] (Fig. 1a). It was previously believed that ACh was secreted to the pulmonic parenchyma exclusively by the vagus nerve, but a recent work by Wessler et al. revealed that ACh is also secreted by non-neuronal cells such as smooth muscle cells and eosinophils [13–15]. Autocrine- or paracrine-derived ACh from non-neuronal cells could potentially play an essential role in the onset and progression of asthma than previously thought. The major source of ACh secretion in asthma will be a valuable information to develop an improved treatment for asthma. A previous study that used M3 mAChR-deficient mice found that ACh and cholinergic defects greatly affected asthma pathogenesis [16] but was not able to shed light on the details of the interactive mechanism linking ACh and asthma severity. Further exploration was not possible because of the difficulties in imaging ACh distribution and abundance in lung tissue, which arise from the extremely low quantities of ACh present in the lungs and the rapid degradation of ACh by acetylcholinesterase (AChE). As a result of these challenges, quantitative studies determining the presence of ACh in the microenvironment of asthmatic lungs remains less.

Previously, our group and other researchers applied matrix-assisted laser desorption/ionisation (MALDI) imaging mass spectrometry (IMS) [17] (Fig. 1b) to visualise neurotransmitters in biological specimens [18–20]. Particularly, we successfully detected and visualised ACh distribution in young mouse brain tissue by using tandem mass spectrometry (MS) analysis [18]. In this study, to further develop the ACh imaging by MS/MS analysis and establish a more improved ACh visualising technique, we employed Fourier transform ion cyclotron resonance mass spectrometry (FT-ICR-MS), which has ultra-high mass resolution and signal/noise (S/N) ratio, for the stable ACh imaging on lung tissues. In addition, to study the effects of asthmatic severity on ACh levels in lungs, we applied the ACh imaging method to the two groups of house dust mite (HDM)-sensitised asthma model mice harbouring different inflammatory levels. Here, we used the label-free ACh imaging technique by FT-ICR-MS and asthma model mice to study and elucidate the significance of secreted ACh as a critical particle connecting inflammation to AHR in asthmatic conditions.

## Materials and methods

### Mice and experimental asthma model

This study was approved by the ethics committee of Hamamatsu University School of Medicine and conducted according to the guidelines for animal experimentation required by this institution. In this study, we used an established asthma model mice sensitised by house dust mite (HDM) and aluminium adjuvant with minor modification as described previously [21]. Female BALB/c mice (8 weeks old) were purchased from Japan SLC (Hamamatsu, Japan). In this study, we prepared two groups of HDM-sensitised asthma model mice harbouring different inflammatory levels. To induce different allergic inflammatory levels in mice, we employed an Alum adjuvant that leads to potent immune responses. Mice were intraperitoneally (i.p.) immunised with HDM (50 μg, GREER, Lenoir, NC, USA) with Alum (2 mg, Al(OH)_3_, Thermo, Rockford, IL, USA) (HDM/Alum group) or intranasally (i.n.) sensitised with 50 μg HDM (HDM group) on day 1. Both groups of mice received 25 μg HDM sensitisation (i.n.) on days 8 and 13. As a normal control, mice were administered saline (Otsuka Pharmaceutical, Tokyo, Japan) (i.n.) instead of the antigen (saline group). The sensitised mice, as well as the saline-infused mice, were subjected to various experiments on day 15. Each experiment described below was performed with 6 mice per group.

### Lung tissue collection and cryosectioning for FT-ICR-MS

Mice were deeply anaesthetised by i.p. injection of ketamine (100 mg/kg) and xylazine (10 mg/kg). Under anaesthesia, the lungs were carefully and quickly inflated with an infusion of 0.5% carboxymethyl cellulose (CMC)/PBS from the trachea, and rapidly dissected and embedded with 4% CMC/PBS followed by immediate freezing in liquid nitrogen. We conducted the lung tissue sampling to all of the mice from death to freezing within 2 min. The frozen lung tissues were stored at − 80 °C until analysis. The frozen lung samples were sliced by a cryostat (CM1950; Leica, Wetzlar, Germany) into 10 μm sections and thaw-mounted on indium tin oxide (ITO)-coated glass slides (MATSUNAMI, Osaka, Japan) at − 20 °C. The lung sections were cut along the long flat frontal plane of the left lobe and stored in small desiccators at − 80 °C until use.

### Mass spectrometry instrumentation

All IMS experiments were performed on a solariX XR 7.0 T FT-ICR mass spectrometer (Bruker Daltonics, Leipzig, Germany). The MS spectra between *m/z* 50–350 were measured in positive ion mode by 500 laser shots per pixel. The ACh distribution was analysed at a 100 μm laser pitch (100 μm spatial resolution).

### Sample preparation for FT-ICR-MS

Immediately after removal from − 80 °C to room temperature (RT), the slide-mounted sections were dried in a vacuum desiccator for at least 30 min at RT. Before the matrix application, the slide samples were optically imaged using a scanner (EPSON, Tokyo, Japan). Next, we sprayed 40 mg/mL 2,5-dihydroxy benzoic acid (DHB, Bruker Daltonics) in 125 mM ammonium sulphate and 50% methanol as a matrix utilising a TM Sprayer (HTX Technologies, Carrboro, NC, USA). The matrix sprayed lung sections and standard spots were FT-ICR-MS analysed following the procedure described below.

### Data acquisition on ACh standards by FT-ICR-MS

For the determination of the experimental value of ACh *m/z* in FT-ICR-MS analysis, we manually spotted a standard solution (1 mg/mL, 0.5 μL/spot) of ACh (Wako, Saitama, Japan) on ITO-coated glass slides (MATSUNAMI, Osaka, Japan) and measured the *m/z* value of ACh. We then analysed the ACh signal on control lung sections (saline group). We compared the obtained signal peaks on the lung tissues with the peaks of the ACh standard, and identified the experimental value of ACh *m/z* on the mice lung sections. To construct a standard curve, we spotted different amounts of ACh (100, 50, 25, 12.5, 6.25, 3.13, 1.56, 0.78, 0.39, 0.20 pmol in 0.2 μL, 0.2 μL/spot) on control mouse lung tissue sections (saline group) on an ITO-coated slide, designated the spots as regions of interest, and measured the ACh signal intensity. The measurement was repeated at eight times per an ACh solution, and we calculated relative standard deviation (RSD) % for evaluating reproducibility. The error bars in the standard curve were made by the repeated and independent experiments (*N* = 8 technical replicates for each experiment). The correlation coefficient was calculated by the standard curve. In the analysis of the dilution series of ACh standards, we imaged ACh amount on the spots of the standard solutions.

### ACh imaging on mouse lung tissues by FT-ICR-MS

For the ACh visualisation on lung tissues by FT-ICR-MS analysis, we simultaneously analysed the three groups of mice. We placed one or two lung sections of the three experimental groups on the same ITO-coated conductive glass slide, and conducted an FT-ICR-MS analysis to at least one section per each group. The lung sections of each mouse were assayed with at least two technical replicates. The order of FT-ICR-MS assays among the three groups was randomised by experimenters in each analysis. Moreover, just before and during the FT-ICR-MS measurements, we calibrated the measuring condition using a DHB-derived ion (*m/z* 155.03389) for the calibration of MALDI to minimise the measurement error between the analyses as an online calibration. After FT-ICR-MS, the same mouse lung sections on slides were subjected to haematoxylin-eosin (HE) staining and imaged by a NanoZoomer (Hamamatsu Photonics, Hamamatsu, Japan) for analysing a correlation between ACh intensities and the histological features of the lung tissues.

### Data analysis for FT-ICR-MS

The measured data of FT-ICR-MS were analysed with flexImaging Software (version 4.1, Bruker Daltonics). The mass filter window of the ACh signal was designated *m/z* 146.117 ± 0.02. In the analysis of the FT-ICR-MS data regarding the ACh standard and the intravital ACh on mouse lung tissues, based on previous studies [22–25], we employed a representative signal of the DHB-derived ions (*m/z* 155.03389) which was near to the ACh signal as an internal standard and normalised the ACh signals dividing the ACh intensity by the DHB intensity in each analysis.

### Measurement of airway hyperresponsiveness

Airway resistance and compliance were evaluated using the Buxco FinePointe RC System (Data Science International, Wilmington, NC, USA), in which mice were mechanically ventilated using a previously described technique with minor modifications [21, 26]. Under deep anaesthetisation, mice were challenged with aerosolised PBS (baseline) followed by increasing doses of methacholine (2.5, 5.0, 10, 20 and 40 mg). Maximum lung resistance (R_L_) and minimum dynamic compliance (C_dyn_) were recorded during a 3-min period after each methacholine challenge.

### Collection of bronchoalveolar lavage fluid, lung tissue fixation and histology

After AHR measurement, the mice trachea were cannulated and the lungs were lavaged three times with 1 mL of ice-cold PBS to collect bronchoalveolar lavage (BAL) cells, as previously described [21, 26]. After bronchoalveolar lavage fluid (BALF) sampling, the mice lungs were transcardially perfused with ice-cold PBS to remove red blood cells, and the lungs were sampled and fixed for histology with 4% paraformaldehyde in PBS. The fixed lung tissue was embedded in paraffin, sliced into several 5 μm sections and histologically analysed by HE and periodic acid-Schiff (PAS) staining. The stained sections were imaged by a NanoZoomer (Hamamatsu Photonics, Hamamatsu, Japan).

### Flow cytometry

BAL cells were stained with allophycocyanin (APC)-labelled anti-Ly-6G/Ly-6C (clone RB6-8C5; BioLegend, San Diego, CA, USA), Alexa Fluor-labelled anti-CD19 (clone 6D5; BioLegend), phycoerythrin (PE)-labelled anti-Siglec-F (clone E50-2440; BD Pharmingen, San Diego, CA, USA), and PE-Cy (PE-Cy7)-labelled anti-CD45 (clone 30-F11; BioLegend), peridinin-chlorophyll-protein complex-Cy5.5 (PerCP-Cy5.5)-labelled anti-CD3e (clone145-2C11; eBioscience, San Diego, CA, USA), eFluor-450-labelled anti-CD11b (clone M1/70; eBioscience) and APC-Cy7-labelled anti-CD11c (clone N418; BioLegend). Flow cytometry was carried out using a FACSCanto II (BD Bioscience, San Jose, CA, USA) and the data were analysed with FlowJo version 8.6 software (TreeStar Inc., Ashland, OR, USA).

### Preparation of lung tissue lysate and assay for acetylcholinesterase activity

For acetylcholinesterase (AChE) activity assay, mice lungs were rapidly dissected under deep anaesthetisation and frozen in liquid nitrogen. The frozen lung samples were homogenised in ice-cold phosphate buffer (50 mM, pH 7.4) using an Ultrasonic Homogenizer VP-050N (TAITEC, Tokyo, Japan). The lung homogenates were centrifuged at 11,000×*g* for 20 min, and the supernatants were aliquoted and stored at − 80 °C until assay. The concentration of total protein was determined using the Quick Start Bradford Protein Assay reagents (Bio-Rad, Tokyo, Japan) and bovine serum albumin standard (Bio-Rad). AChE activity was measured by duplicate analysis per sample using the Amplite Fluorimetric Acetylcholinesterase Assay Kit (AAT Bioquest, Sunnyvale, CA, USA) in accordance with the manufacturer’s instructions. The AChE activity of each sample was determined by the standard curve, and enzymatic activity was calculated as mU/mg protein.

### Statistical analysis

Unpaired two-tailed Student’s *t* tests were used for comparisons between groups. *P* values less than 0.05 were considered statistically significant. All data are represented as means ± standard error of the mean (SEM). The number of biological replicates in each experiment was 6 mice per group for ACh imaging by FT-ICR-MS, 6 mice per group for asthma physiological phenotyping and histology, and 6 mice per group for AChE activity assay (as noted in the figure legends).

### Data availability statement

The datasets generated and/or analysed during the current study are available from the corresponding author on reasonable request.

## Results

### Acetylcholine signal detection and the validation on mouse lung tissue

**Fig. 2:**
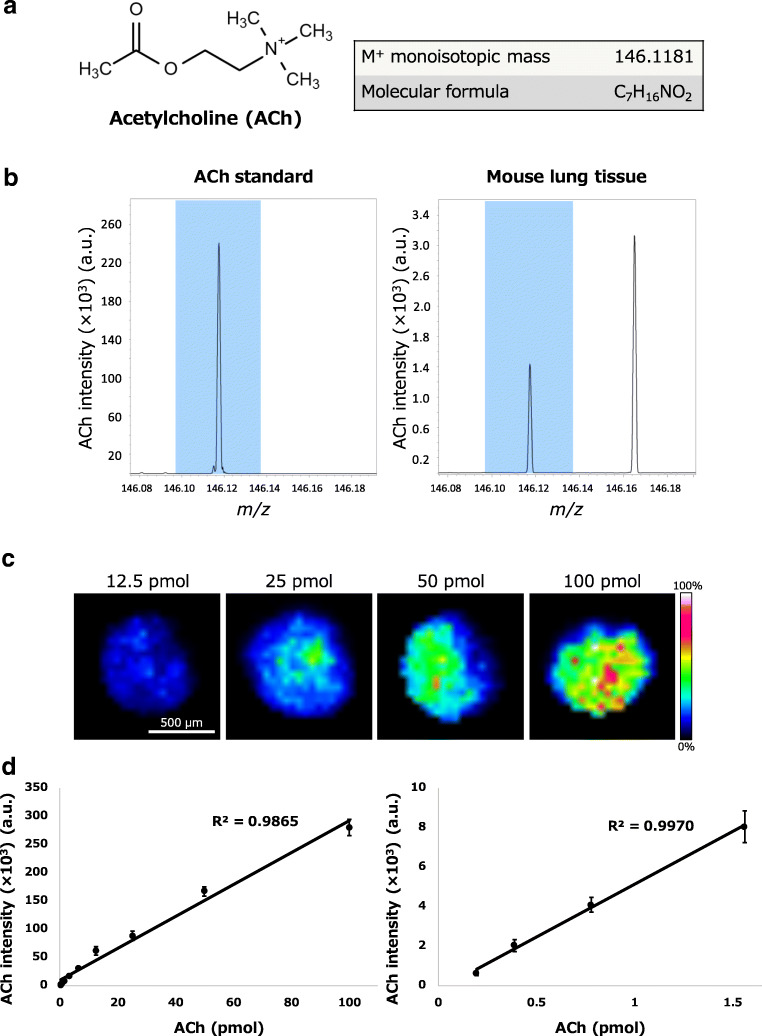
ACh signal and amount-dependent signal intensity. **a** Chemical structure of ACh. The theoretical exact mass of ACh is 146.1181 (C_7_H_16_NO_2_, M^+^ monoisotopic mass). **b** Comparison of ACh signal peaks between the standard and lung tissue. The *m/z* value of both ACh signals coincided at 146.117. **c** The ACh abundance in spots was visualised by Fourier transform ion cyclotron resonance mass spectrometry (FT-ICR-MS) of the dilution series of standards on lung sections. The ACh images were reflected by ACh amount of the ACh standard solutions. **d** Ion signal linearity. The standard curve was constructed from the values of the FT-ICR-MS analysis. The linearity of standard curves on all range (left) and on low ACh concentration (right) is shown. The error bars in the standard curve were made by the repeated and independent experiments (*N* = 8 technical replicates for each experiment). The data of ACh standard on lung tissues yielded the linear correlation coefficient (*R*^2^ = 0.9865 in all range and *R*^2^ = 0.9970 in low concentration range). Scale bar 500 μm. Some of the error bars are less than the size of the markers. Data are shown as mean ± SEM. a.u., arbitrary unit

ACh (Fig. 2a) exists intracellularly and extracellularly in the mammalian lung tissue. To distinguish the ACh signal from matrix-derived noise in the mouse lung tissue, we utilised ultra-high mass resolution FT-ICR-MS. It is already known that IMS laser irradiation in positive ionisation mode generates positively charged ACh ions (C_7_H_16_NO_2_, theoretical mass value 146.1181) by charging at the N^+^ site without protonation (Fig. 2a). Therefore, we initially determined the exact *m/z* value of ACh by measuring a spot of ACh standard solution on an ITO-coated slide. The ACh signal peak emerged at *m/z* 146.117 without matrix-derived noise around the *m/z* 146.120 mass window (*m/z* 146.117 ± 0.02) (Fig. 2b). Next, we targeted the intravital ACh signals in mouse lung sections. In the analysis of the lung sections, we successfully detected the ACh signals by FT-ICR-MS in the mouse lung tissue as well as in the ACh standard spot, as shown in Fig. 2b. The exact *m/z* value of the ACh signal peaks were identical between the ACh standard and mouse lung section. The nearest peak in the immediate vicinity of the ACh signal was detected at *m/z* 146.163 in the mouse lung tissue but not in the standard. This result indicates that the noise peak (*m/z* 146.163) was derived from the mouse lung tissue, not from the 2,5-dihydroxybenzoic acid (DHB) matrix, thereby demonstrating that the difference between the ACh signal and tissue-derived noise can be clearly discriminated by utilising extremely high mass resolution FT-ICR-MS.

We then evaluated the validity of the imaging results, specifically whether the ACh signal intensity and accompanying graphical images depended on the ACh amount in the FT-ICR-MS measurement, by using a dilution series of the ACh standard solution. We spotted the ACh dilutions (100, 50, 25, 12.5, 6.25, 3.13, 1.56, 0.78, 0.39, and 0.20 pmol in 0.2 μL/spot) on the control mouse’s lung tissue sections at an ITO-coated slide, designated the spots as the regions of interest, and measured the ACh signal intensity. The abundance of ACh in standard spots on lung sections was imaged at a spatial resolution of 100 μm (shown in Fig. 2c), and the images revealed that the signal intensity and graphical images were highly correlated with the ACh amount. According to the previous studies [22–25], the FT-ICR-MS data of the ACh standard on lung tissues were normalised by a representative DHB-derived ion signal (*m/z* 155.03389) as an internal standard. This normalisation was performed for the compensation for inhomogeneous MALDI matrix coating, differential ionisation efficiencies, and crystal heterogeneity in the FT-ICR-MS analysis. After the normalisation with the internal standard, we generated the standard curve from the ACh standard data (shown as Fig. 2d). From the calculated standard curve, we obtained a high correlation coefficient on all ranges (*R*^2^ = 0.9865, from 100 to 0.20 pmol) and low ACh concentration range (*R*^2^ = 0.9970, from 1.56 to 0.20 pmol) regarding the ACh standards using the FT-ICR-MS. The FT-ICR-MS measurements revealed that the standard curve had a linear relationship between the ACh intensity and the amount from 0.20 to 100 pmol. These results confirmed that this technique is capable of imaging ACh distribution in mouse lung tissue in an amount-dependent manner.

We also calculated relative standard deviation (RSD) % for evaluating reproducibility of the FT-ICR-MS measurements (Table 1). The RSD% value of the ACh intensity ranged from ~ 15% on high ACh concentration (100, 50 pmol) and 25–35% on middle ACh amount (25, 12.5, 6.25, 3.13, 1.56, 0.78 pmol) to approximately 45% on low ACh concentration (0.39, 0.20 pmol). The FT-ICR-MS showed a robust reproducibility on a high ACh amount, but the reproducibility was relatively decreased on the low amount of ACh.

**Table 1:** Analysis of relative standard deviations (RSD) %

Standard	ACh (pmol)									
	0.20	0.39	0.78	1.56	3.13	6.25	12.5	25	50	100
Intensity (× 10^3^) (a.u.)	0.61 ± 0.10	2.03 ± 0.31	4.08 ± 0.38	8.05 ± 0.80	16.04 ± 1.91	30.58 ± 2.70	61.57 ± 7.11	88.20 ± 8.00	167.20 ± 8.36	280.27 ± 14.54
RSD% (%)	46.55	43.09	26.17	28.17	33.72	24.95	32.67	25.65	14.14	14.67

Data are shown as mean ± SEM. *N* = 8 technical replicates for each experiment. *a.u.*, arbitrary unit

### Preparation of asthma model mice

**Fig. 3:**
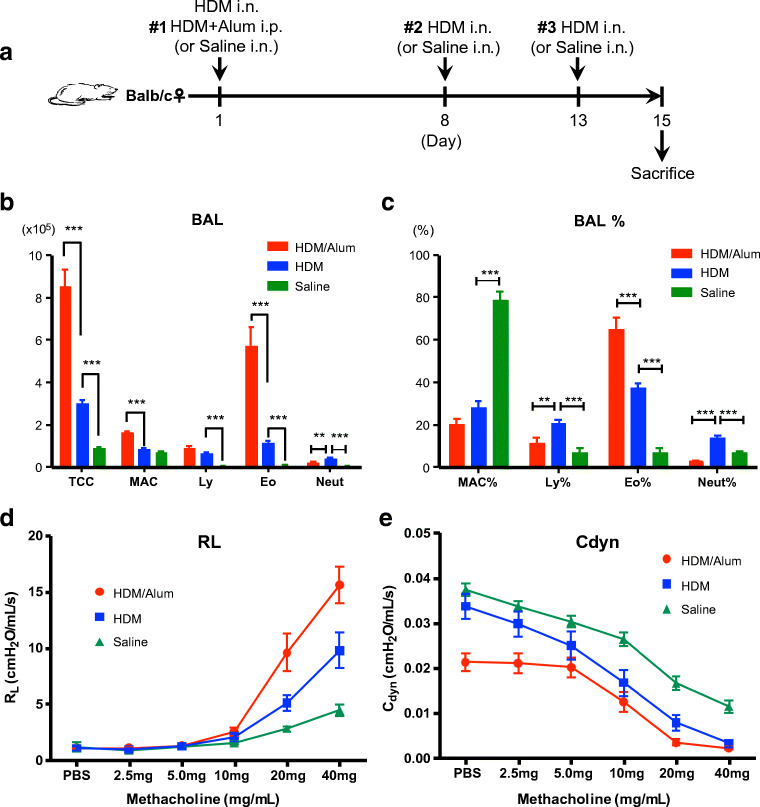
Experimental timeline and asthma physiological phenotypes. **a** Experimental timeline. Three experimental groups were prepared in this study by treatment with saline, house dust mites (HDM), or HDM/Alum. We employed HDM as a sensitising antigen to induce asthma-like pathophysiology, and the allergic response was potentiated by injection of Alum adjuvant. **b** Differential cell counts in bronchoalveolar lavage fluid (BALF). **c** Percentage of differential cell counts in BALF. **d** Lung resistance (R_L_) in airway hyperresponsiveness (AHR). **e** R_L_ in dynamic compliance (C_dyn_). Lung physiological reactions against methacholine were measured on day 15. Data are shown as mean ± SEM (*N* = 6 mice per group). *P < 0.05, **P < 0.01 and ***P < 0.001. i.n., intranasal; i.p., intraperitoneal; TCC, total cell count; MAC, macrophages; Ly, lymphocytes; Eo, eosinophils; Neut, neutrophils; PBS, phosphate-buffered saline

We utilised an already established asthma rodent model induced by several antigen sensitisation as an asthmatic animal model [21]. To induce human-like asthma, the mice were repeatedly sensitised with HDM or Alum-adjuvanted HDM as an antigen for 2 weeks (Fig. 3a). For the validation of the onset of asthmatic pathology, we analysed the number of inflammatory cells in bronchoalveolar lavage fluid (BALF) obtained from the lungs of mice (Table 2, Fig. 3b, c). The inflammatory cell count was significantly higher in the BALF of HDM-sensitised mice than that in saline-infused mice (HDM 3.03 ± 0.15 (× 10^5^) cells; saline: 0.90 ± 0.05 (× 10^5^) cells; *P* < 0.001) (Table 2, Fig. 3b), and HDM/Alum-sensitised mice had a higher number of inflammatory cells (HDM/Alum 8.52 ± 0.83 (× 10^5^) cells; vs. HDM; *P* < 0.001). Thus, cellular infiltration to the lung airways occurred extensively in the HDM-infused mice and even more so in HDM/Alum-infused mice. In particular, the number of eosinophils in the BALF of HDM-sensitised mice was notably increased relative to non-sensitised mice (HDM 1.15 ± 0.11 (× 10^5^) cells; saline 0.06 ± 0.02 (× 10^5^) cells; *P* < 0.001). Furthermore, eosinophil recruitment in HDM/Alum-sensitised mice was approximately fourfold greater than that in HDM-sensitised mice (HDM/Alum 5.74 ± 0.87 (× 10^5^) cells; vs. HDM; *P* < 0.001). Eosinophils accounted for the largest proportion of cells in BALF from the two asthma model mice, whereas macrophages were the primary cell type in BALF from the control mice (Table 2, Fig. 3c). The eosinophil percentages were 65.2 ± 5.24% and 37.4 ± 2.09% in the HDM/Alum and HDM groups, respectively. Moreover, the eosinophil percentage was just 7.16 ± 1.83% in the saline group (*P* < 0.001, HDM/Alum vs. HDM; HDM vs. saline). These results revealed that both asthmatic mouse models harboured the eosinophilic asthma phenotype, and Alum-adjuvanted HDM induced more advanced eosinophilic inflammation. Thus, we indeed prepared two asthmatic mouse models with different inflammation levels. We also examined AHR and dynamic compliance (C_dyn_) of the lungs of these mice (Table 3, Fig. 3d, e). AHR measurement revealed that the HDM group showed significantly increased hypersensitivity in the 20.0 mg and 40.0 mg methacholine challenges (HDM 11.7 ± 3.05 cmH_2_O/mL/s; saline 4.10 ± 0.46 cmH_2_O/mL/s; *P* < 0.01, in 40.0 mg challenges) (Table 3, Fig. 3d), and responsiveness was further elevated in the HDM/Alum group by methacholine stimuli (HDM/Alum 15.6 ± 1.64 cmH_2_O/mL/s; vs. HDM; *P* < 0.05, in 40.0 mg challenges). These results revealed that the level of hyperreactivity matched the level of eosinophilic inflammation. In addition, C_dyn_ measurement showed that the compliance of the HDM group was significantly decreased compared with the saline group for the methacholine challenges (*P* < 0.05, in 10.0 mg; *P* < 0.01, in 20.0 mg; *P* < 0.001, in 40.0 mg challenges) (Table 3, Fig. 3e), with the HDM/Alum group exhibiting further decline in compliance (*P* < 0.05, in 2.5 mg; *P* < 0.01, in PBS and 20.0 mg challenges). Notably, we observed a decrease in airway compliance even with PBS exposure in HDM/Alum-sensitised mice, thereby suggesting that they have persistent and strong airway constriction. Furthermore, this deteriorative trend in compliance correlated with the worsening of inflammatory responses in the lungs of two types of the HDM-challenged mice. Thus, we found that the C_dyn_ of lungs was also impaired in HDM-challenged mice, and adjuvant-combined HDM exposure severely damaged lung retractility in mice. These findings also verified HDM-sensitised and HDM/Alum-sensitised mice as an asthma model and severe asthma model, respectively. We then proceeded to the FT-ICR-MS analysis by using their lung samples.

**Table 2:** Analysis of inflammatory cells in bronchoalveolar lavage fluid (BALF)

BALF (BALF %)	Saline (× 10^5^ cells) (%)	HDM (× 10^5^ cells) (%)	HDM/Alum (× 10^5^ cells) (%)
Total cell count	0.90 ± 0.05	3.03 ± 0.15***	8.52 ± 0.83***
Macrophages	0.71 ± 0.06	0.83 ± 0.05	1.64 ± 0.05***
	(78.8 ± 4.02)	(28.1 ± 2.90***)	(20.4 ± 2.54)
Lymphocytes	0.06 ± 0.02	0.64 ± 0.07***	0.90 ± 0.10
	(6.96 ± 1.86)	(20.7 ± 1.50***)	(11.4 ± 2.43**)
Eosinophils	0.06 ± 0.02	1.15 ± 0.11***	5.74 ± 0.87***
	(7.16 ± 1.83)	(37.4 ± 2.09***)	(65.2 ± 5.24***)
Neutrophils	0.06 ± 0.003	0.41 ± 0.04***	0.23 ± 0.02**
	(7.05 ± 0.50)	(13.7 ± 1.23***)	(2.87 ± 0.47***)

Data are shown as mean ± SEM (*N* = 6 mice per group). Asterisks mean significance values for HDM vs. saline or HDM/Alum vs. HDM. **P < 0.01, ***P < 0.001

**Table 3:** Analysis of airway hyperresponsiveness (AHR)

AHR	Group	PBS	Methacholine challenge (mg)				
			2.5	5.0	10	20	40
R_L_ (cmH_2_O/mL/s)	Saline	1.19 ± 0.38	0.90 ± 0.02	1.22 ± 0.06	1.55 ± 0.11	2.81 ± 0.23	4.10 ± 0.46
	HDM	1.06 ± 0.16	0.94 ± 0.08	1.23 ± 0.15	2.03 ± 0.39	5.08 ± 0.71**	11.7 ± 3.05**
	HDM/Alum	1.07 ± 0.06	1.08 ± 0.05	1.28 ± 0.08	2.55 ± 0.36	9.57 ± 1.69*	15.6 ± 1.64*
C_dyn_ (cmH_2_O/mL/s)	Saline	0.037 ± 0.001	0.034 ± 0.001	0.030 ± 0.001	0.026 ± 0.002	0.017 ± 0.001	0.011 ± 0.001
	HDM	0.034 ± 0.003	0.030 ± 0.003	0.025 ± 0.003	0.018 ± 0.003*	0.008 ± 0.002**	0.003 ± 0.001***
	HDM/Alum	0.021 ± 0.002**	0.021 ± 0.002*	0.020 ± 0.002	0.012 ± 0.002	0.003 ± 0.001**	0.002 ± 0.001

Lung resistance (R_L_) and dynamic compliance (C_dyn_). Data are shown as mean ± SEM (*N* = 6 mice per group). Asterisks mean significance values for HDM vs. saline or HDM/Alum vs. HDM. *P < 0.05, **P < 0.01 and ***P < 0.001

### ACh imaging by FT-ICR-MS on the lungs of asthma model mice

**Fig. 4:**
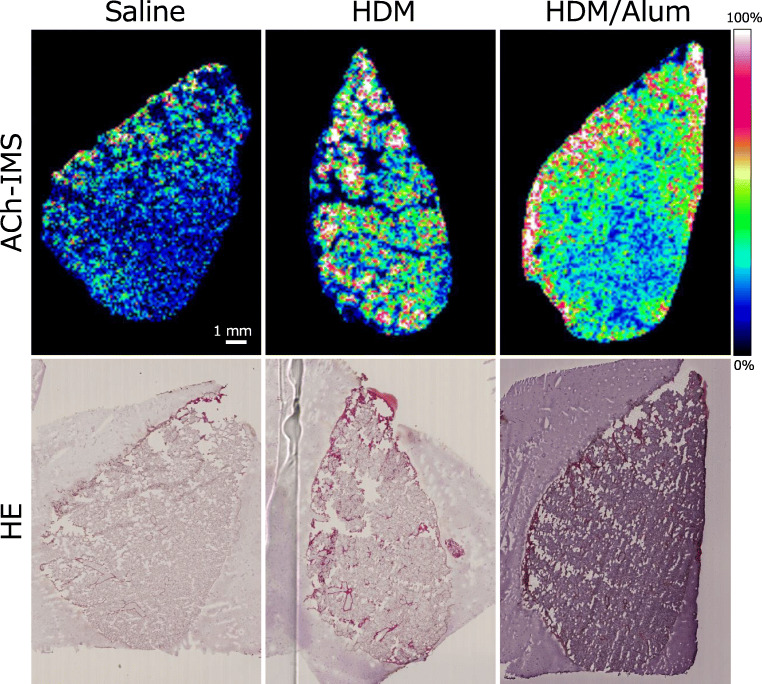
ACh elevation with aggravation of asthma. Representative ACh distribution images (top panels) and HE-stained lung tissues (bottom panels). These imaging data show that ACh abundance in the lungs of HDM-sensitised mice was remarkably elevated compared with saline-infused control mice and even further elevated in HDM/Alum-sensitised mice. Scale bar 1 mm

**Fig. 5:**
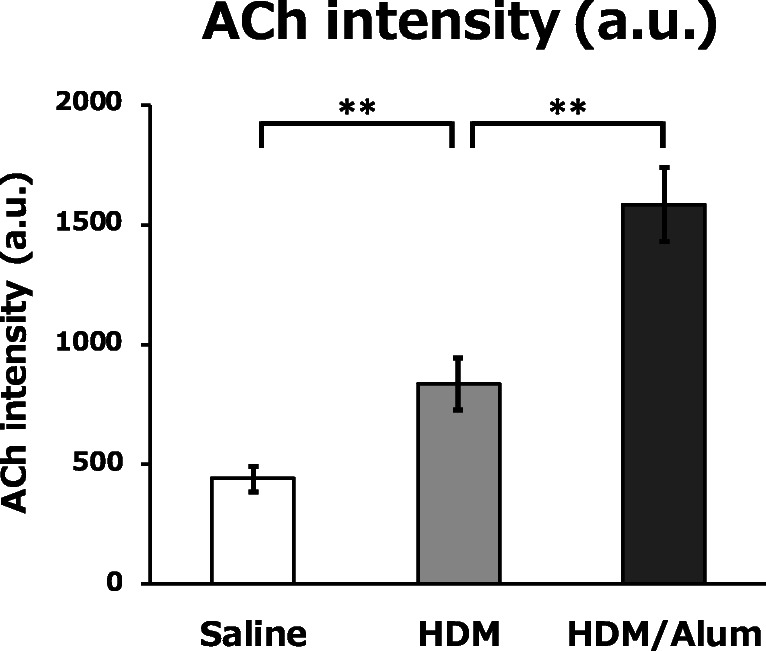
Quantitative analysis demonstrates ACh increase with asthma severity. The quantitative analysis of ACh intensity per lung section revealed that the localised ACh quantity in the lungs of HDM-sensitised mice was significantly higher than that of saline-infused mice and even higher in the lungs of HDM/Alum-sensitised mice. ACh signal intensity was doubled in the HDM group and tripled in the HDM/Alum group relative to the saline group. The lung sections of each mouse were analysed with at least two technical replicates. Data are shown as mean ± SEM (*N* = 6 mice per group). **P < 0.01. a.u., arbitrary unit

We applied the ACh imaging with FT-ICR-MS in lung tissue samples from the three mouse groups to visualise the cholinergic phenotype in asthma. In the analysis, according to the previous studies [22–25], we also normalised each FT-ICR-MS data on intravital ACh with a DHB-derived ion (*m/z* 155.03389) as an internal standard for the compensation of the subtle heterogeneity of the measuring conditions between laser irradiation and lung sections. Using the ACh imaging technique, we analysed the lung tissues of six mice per group as shown in the representative images of ACh distribution in lungs from each animal in Fig. 4 and Electronic Supplementary Material (ESM) Fig. S1. As shown in Fig. 4 and ESM Fig. S1, we successfully imaged both ACh abundance and distribution in the mouse lung tissue, which showed clear differences between the experimental groups. Intriguingly, the lungs of saline-infused mice showed a nearly uniform distribution of ACh with sparse spots of concentrated ACh (yellow-green spots in Fig. 4 and ESM Fig. S1). The lungs of HDM-sensitised mice showed significantly increased ACh intensity than the lungs of saline-infused mice, and the lungs of HDM/Alum-sensitised mice showed the greatest abundance of ACh. These ACh distribution images showed the distribution pattern of ACh by a heat map corresponding to the ACh amount (ACh levels indicated by colour, black: not detected; blue: low; yellow-green: medium; yellow: high; red: ultra-high; and white: extremely high). The FT-ICR-MS analysis revealed that the asthma model groups displayed a similar increasing tendency of ACh abundance, whereas there was a modest individual difference of the ACh distribution pattern between animals in an experimental group. Quantitative analysis of the averaged ACh signal intensity per entire lung section also showed that ACh quantity was significantly elevated in the lungs of HDM-challenged mice compared with saline-infused mice (HDM 835 ± 110; saline 440 ± 53; *P* < 0.01, saline vs. HDM) (Fig. 5) and further elevated in the lungs of HDM/Alum-challenged mice (HDM/Alum 1586 ± 154; *P* < 0.01, HDM vs. HDM/Alum). The average ACh intensities and the standard curve indicated that the ACh amount on lung in the HDM group and the HDM/Alum group was nearly doubled and tripled compared with the saline group, respectively. Moreover, these intensity results strongly correlated with the aggravation of asthmatic airway hypersensitivity induced by HDM. These results indicated that ACh quantity in the lungs was significantly involved with the physiological responses induced by the antigen sensitisation and the Alum adjuvant infusion in the asthma model animals. Thus, the ACh data were in accordance with the severity level of asthma-like airway malfunctions.

### Histological and biochemical analysis of the lungs of asthma model mice

**Fig. 6:**
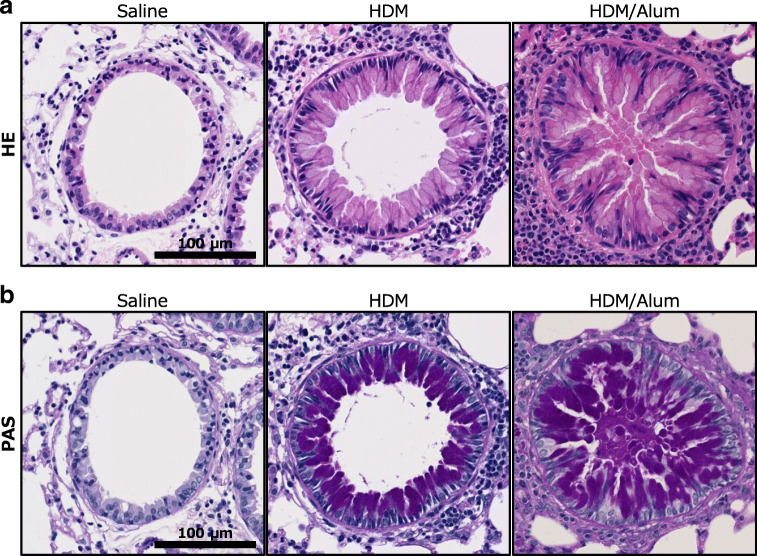
Bronchial constriction in the airways of asthma model mice. Representative airway images. **a** Haematoxylin-eosin (HE)-stained lung sections. The airways of asthma model mice showed smooth muscle hypertrophy and partial airflow obstruction. **b** Periodic acid-Schiff (PAS)-stained lung sections. Hyperplasia of mucous goblet cells was observed in the asthma model mice. The HDM-sensitised mice showed narrowing of airways, and some airways were severely obstructed in the HDM/Alum-sensitised mice. Alum-adjuvanted HDM sensitisation strongly aggravated asthma bronchoconstriction. Scale bars 100 μm

To determine the leading cause of ACh elevation in the lungs of asthma model mice, we further investigated their lung tissues by histological and biochemical assays. To histologically examine the inflammatory changes, we first observed the pathologic states of the bronchioli scattered throughout the lung sections by performing haematoxylin-eosin (HE) and periodic acid-Schiff (PAS) staining. By HE staining, we found that the two asthma mice models had a thickened external layer surrounding the airway compared with the control mice (Fig. 6a), thereby indicating that HDM sensitisation induced substantial inflammation in the airway components throughout the lung. PAS staining revealed that the lungs of the HDM group harboured asthma-like lesions that are characterised by dysplasia of excessive goblet cells (Fig. 6b) and that the injection of Alum adjuvant promoted the pathogenic process. The histological results revealed that the airways of the two asthma models were severely narrowed and the respiratory epithelia were abnormally hypertrophied compared with controls. Furthermore, severe airway obstruction was observed in some bronchioli of the HDM/Alum group, which was caused by hyperplastic goblet cells secreting mucosal fluid, as shown in Fig. 6 b. The hypertrophied airway epithelium and goblet cells were strongly stained violet by PAS staining in the HDM and HDM/Alum groups, thereby indicating that they secreted substantial mucosal fluid into the airways. The airway constriction in the asthma models was observed throughout the entire lung sections; thus, these data indirectly supported the observations for the increasing tendency of the ACh amount in the asthmatic lungs. Altogether, these histological alterations indicated that the airways of asthma model mice experienced intense inflammatory reactions due to repeated sensitisation and formed remodelling-like airway lesions. These levels of the airway constriction correlated well with the increasing tendency of the ACh amount in their lung tissues.

**Fig. 7:**
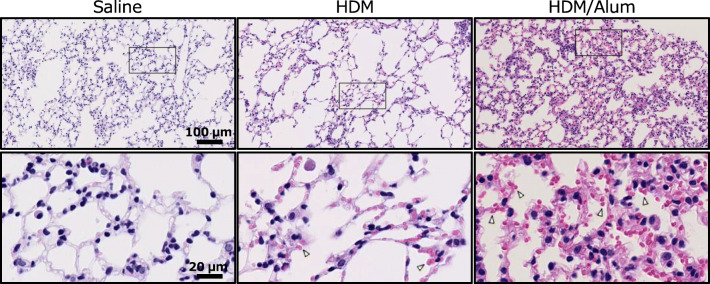
Eosinophil infiltration into the lung parenchyma of asthma model mice. Micrographs of representative lung parenchyma with haematoxylin-eosin (HE) staining. Representative bronchioli and alveoli in the lung are displayed for each experimental group. Yellow arrowheads indicate the infiltrated eosinophils (eosin-stained cells, red). The HDM-sensitised mice had eosinophils in the lung subcomponents, whereas almost no eosinophils were observed in the lung subcomponents of saline-infused mice. The HDM/Alum-sensitised mice displayed the most intense eosinophil invasion into the lung airways. The lower panels are magnified images of the windows indicated in the upper panels. Scale bars 100 μm (above) and 20 μm (below)

To identify the detailed inframammary reactions on lung parenchyma at a more micro-scale level, we analysed the airway of the asthma models in the magnified view. HE staining further showed that the bronchioli and alveoli of the HDM and HDM/Alum groups harboured the infiltration of inflammatory eosinophilic cells (Fig. 7, red, indicated by arrowheads). The infiltrated eosinophils were scattered in an almost uniform manner throughout the entire lung, and the eosinophilic infiltration appeared to be further exacerbated in the HDM/Alum group relative to the HDM group. Notably, the distribution pattern and quantitative changes of invading eosinophils matched well with the ACh behaviour observed by the ACh imaging in the lungs of the asthma model animals. Thus, these inflammatory responses such as the thickened external layer around airway, the hypertrophied goblet cells and the eosinophil infiltration were highly likely involved in the elevation of ACh in the lungs of the asthma mice models.

**Fig. 8:**
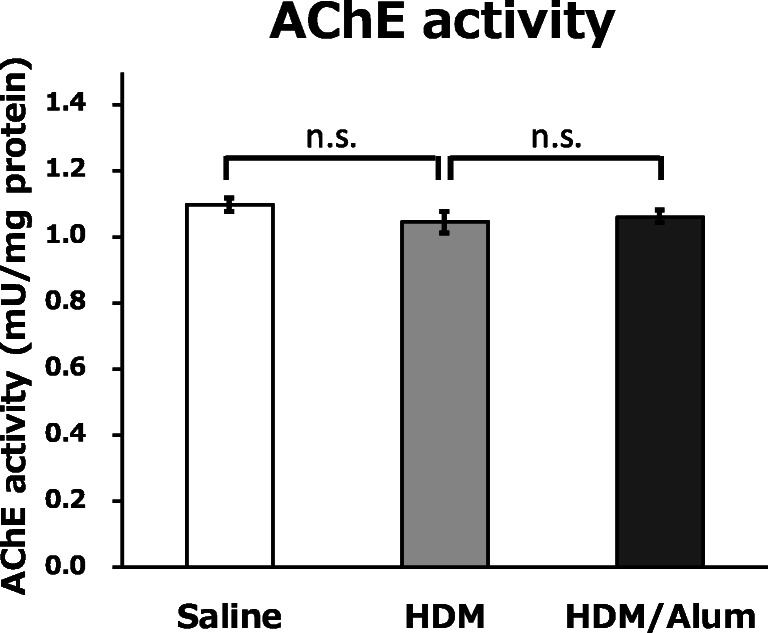
AChE activity did not change with asthma severity. We analysed AChE enzymatic degradative activity using lung homogenate samples. The biochemical measurements showed no significant differences in lung AChE activity between the experimental groups. These results revealed that ACh degradation by AChE was functionally maintained in the asthma model mice. These results indicated that the ACh increase in the lung of the asthma model mice was not induced by the functional deficits of AChE. Data are shown as mean ± SEM (*N* = 6 mice per group). n.s., not significant

To determine the alternations of ACh turnover by related enzymes in lung tissues, we assayed AChE enzymatic degradative activity by using lung homogenate samples. Unexpectedly, there were no significant differences in the AChE activity of lungs between the three experimental groups (Fig. 8). These results revealed that AChE maintained ACh degradative activities in the lungs of the asthma model animals, thereby implying that the increase of ACh in lungs was not induced by a decline in AChE activity but by other factors involved in the asthma inflammatory responses.

## Discussion

Here, by applying FT-ICR-MS, we successfully visualised the abundance and spatial distribution of ACh in the rodent pulmonary tissue (Figs. 1, 2 and 4, and ESM Fig. S1). Ultra-high mass resolution FT-ICR-MS more strictly discriminates ACh peaks from non-target peaks compared with the conventional IMS methods. ACh remains one of the most challenging molecules to directly image, and lungs are also a particularly difficult tissue for imaging because of their vulnerability and dynamic retractility. Indeed, almost all in vivo imaging techniques, including computed tomography, single photon emission computed tomography, positron emission tomography, near-infrared spectroscopy, magnetic resonance imaging and magnetic resonance spectroscopy, still face extreme difficulty in visualising low-molecular mass biomolecules such as ACh in the lungs. A recent study reported an optical ACh imaging method that used specific antibodies in fixed lung tissues [27]. However, the immunohistochemical approach mainly targets intracellular ACh within ACh-synthesising cells, whereas the FT-ICR-MS approach can comprehensively measure both intracellular and extracellular ACh. Additionally, asthma lesions generally exist heterogeneously across the lungs, and the alternative visualisation technique described here allowed us to observe ACh distribution on the plane of the whole lung tissue in a non-biased manner.

We found that ACh was increased in the lungs of HDM-sensitised mice relative to non-sensitised mice, and intriguingly, ACh was further elevated by stronger HDM sensitisation with the adjuvant (Figs. 4 and 5, ESM Fig. S1). Contrary to our expectations, ACh distribution patterns in asthma-like pathologic lungs were nearly uniform but had relatively heterogeneous localisation (Fig. 4, ESM Fig. S1). Mammalian lung tissue consists of innumerable bronchioli and alveoli and receives dense projections from the cholinergic neurons of the vagus nerve. Thus, the observed distribution patterns in the rodent asthma model suggest that ACh is secreted in low concentrations to the entire lung regardless of its central or peripheral location in a normal state. Moreover, its abundance is enhanced by asthma pathology. In addition, we observed intense eosinophil recruitment in the lung in a nearly uniform manner (Fig. 7), which raised the possibility that eosinophils mediated local ACh abundance. The HDM-sensitised mice showed significantly increased AHR and airway narrowing, which suggests that the HDM group had asthma-like functional disturbances in their lungs. Moreover, an Alum adjuvant potentiated the inflammatory responses that resulted in serious lung vulnerability to the methacholine challenge; partial airflow obstruction is often observed in patients with advanced asthma. Indeed, the lungs of the Alum-adjuvanted HDM group exhibited substantially higher levels of inflammation and airway obstruction than the saline and HDM groups, thereby indicating that this group is similar to a severe asthma model (Figs. 3, 6 and 7). The HDM-challenged asthma model mice harboured the eosinophilic asthma phenotype in accordance with previous studies [21, 28, 29]. Eosinophilic asthma provokes T helper 2 (Th2)-induced inflammation [30], which indicates that Th2-type inflammation and elevated eosinophils are closely involved with an increase in ACh. Here, Th2-type inflammation was observed in almost all the asthma model animals [31]. Thus, other asthma model animals with Th2-type inflammation may show similar ACh elevation, and this possibility should be explored in future research. Our results suggest that HDM-induced inflammation especially increases eosinophils and Th2-associated inflammatory cellular responses, further leading to ACh elevation in the airways of the lungs. The quantitative distribution of ACh in the lung tissue was strongly associated with asthma severity, which implies that inflammatory cell levels potentially affect ACh levels in asthma pathology.

The exact mechanism underlying gradual ACh upregulation associated with the progress of allergic inflammation remains unclear. Intravital ACh in the lungs is dually secreted by neuronal vagal nerves and non-neuronal adjacent inflammatory-related immune cells including eosinophils [13–15, 32]. Our results showed that eosinophils extensively infiltrated BALF and lung parenchyma (Figs. 3 and 7), which was consistent with the ACh distribution patterns and elevation trends. These data highlight the possibility that ACh is secreted by eosinophils. Moreover, our results indicated that the AChE in the lungs of the asthma model animals maintained the equivalent ACh degradative activities relative to the normal mice (Fig. 8). These findings suggest that the increase of ACh in lungs was not induced by the functional deficits of the AChE activity but by other asthma inflammatory responses containing excessive ACh secretion mainly mediated by eosinophils. In our study, we sampled and froze the lung tissue approximately 48 h after the last HDM challenge. This suggests that elevated ACh levels persisted 2 days after the last sensitisation and were still too high to be managed by AChE activity. This potential ACh regulation mechanism is consistent with the previous reports stating that ACh secretion was increased by inflammatory mediators (e.g., tachykinins, prostaglandins, thromboxane A_2_) that activate the vagus nerve [33] and eosinophils and major basic proteins blocked the M2 AChRs responsible for autoinhibition of ACh release [34]. In previous asthma studies, AChE activity levels were quite sensitive to the sensitisation protocols and model types [11, 35, 36]; therefore, ACh changes may occur by a slightly different mechanism in other asthma model animals. The present FT-ICR-MS technique is unable to distinguish whether the origin of ACh was intracellular or extracellular and cannot identify the different types of secretory cells. Therefore, further research is required to determine the cellular factors associated with ACh hypersecretion.

In the early stage of the study, we had expected that ACh might be specifically distributed in the lung subregions from central airway to peripheral airway with some concentration gradient in lung tissues and that the ACh distribution manner might be changed in pathologic asthma-like states. Contrary to the expectations, the FT-ICR-MS analysis revealed that the intravital ACh existed in a relatively uniform manner over the lung regardless of central or peripheral airway and the distribution pattern was not significantly altered even in severe asthmatic conditions. Indeed, the FT-ICR-MS did not show the subregion-specific distribution of ACh in lung tissues; however, our study will be the pioneer study that visualised ACh on lung tissues with FT-ICR-MS because almost no study have been performed that measured the ACh existence and distribution in lung tissues by using MALDI-IMS approach and there has been little data about ACh distribution in lungs for many decades. The heterogeneous but relatively uniform ACh distribution within the lungs, even in the asthmatic status, showed by the FT-ICR-MS technique implied that ACh, which is one of the crucial molecules in the asthma pathology, had existed in the lung tissue without significant intraorgan congestion and sparsity. The wide distribution of the intravital ACh in lung suggests that ACh may considerably affect the entire lung, especially in asthmatic conditions. Furthermore, the strong increase of the widely spread ACh throughout the lungs indicates that the drugs that reach and act at peripheral airways are preferable for asthma treatment. The FT-ICR-MS technique may be useful for the efficient screening and evaluations of potential drugs to test whether it works on the peripheral ACh in lung. The advantage of ACh imaging with FT-ICR-MS is that this technique can simultaneously measure both spatial distribution and quantity of the target molecules, whereas the analysis with homogenate samples loses the data of spatial localisation. We believe that the distribution information of ACh in lungs reported by this study cannot be revealed by a conventional bulk tissue analysis such as biochemical assays and liquid chromatography. Moreover, this information will be valuable for elucidating the pathologies of asthma and other pulmonary disease in detail.

FT-ICR-MS for ACh imaging in animal lung tissue still has several technical limitations. Each lung section in the asthma model mice exhibited a unique distribution pattern of ACh. We obtained a good linearity on low ACh concentration range (*R*^2^ = 0.9970) as well as on all ranges (*R*^2^ = 0.9865) in the standard curve and enhanced the imaging quality by processing data with the internal standard and normalisation. However, we could not visualise the characteristic ACh distribution within the bronchioli at single cell resolution to identify the ACh secreting cells in lung. This was because we only succeeded in obtaining clear ACh detection with a good S/N ratio at more than 100 μm laser pitch (spatial resolution of 100 μm) and the ACh intensity fell below the measurable limit due to signal decline with a reduced laser focus. Of note, the ACh detection was still hard even using FT-ICR-MS with ultra-high mass resolution due to the limiting conditions that ACh is extremely low within lung and is a low-molecular compound that is easily blended by the matrix-derived noise peaks. Moreover, in addition to the fragility of the non-fixed lung tissue sections in cryosectioning, tissue damages by laser irradiations during FT-ICR-MS mildly damaged the original substructure of the lung sections, and this destruction made analysis of the minute details between ACh distribution and lung subcomponents more difficult. The FT-ICR-MS revealed that high ACh intensity was apparently observed on the outer edges of the lung section (Fig. 4, ESM Fig. S1). Our results indicated that the ACh accumulation was presumably led by the thaw-mount procedure in cryosectioning or ACh recrystallisation in matrix spraying for ACh liquefaction on sections. We investigated the limit of detection (LOD) by using various concentrations of ACh standard solutions (Table 1, Fig. 2). The FT-ICR-MS analysis showed clear ACh intensity peaks from 100 to 0.20 pmol ranges, but it hardly detected the signal peaks of 0.10 pmol ACh. Thus, the LOD of the FT-ICR-MS can be considered to be 0.20 pmol ACh in 0.2 μL spot. We also evaluated the limit of quantification (LOQ) by calculating RSD% (Table 1). The RSD% value suggests that the reproducibility of the FT-ICR-MS was robust on relatively high ACh concentration range, but it was decreased in low ACh concentration range. Therefore, the ACh data by FT-ICR-MS analysis may be unsuitable for absolute quantification of ACh; however, it can be useful for a comparison analysis with the relative quantitation of ACh amount between the several groups.

Based on previous studies [22–25], we simply normalised the ACh signal by using a representative DHB-derived ion peak (*m/z* 155.03389) as an internal standard in this study. After the FT-ICR-MS data were normalised with the internal standard, we still detected the ACh signal inside the airway surrounded by the epithelial cells of alveoli and bronchioli. The imaging results within the airway seem to indicate that the extracellularly secreted ACh by neuronal cells and non-neuronal cells in lung was detected at the middle of the airway. In the cryosectioning of the lung tissues, tissue cracks unavoidably occurred due to the fragility of the non-fixed lung tissues. In some parts of the non-tissue areas within the micro clefts and streaky tissue ruptures on lung sections, we also detected the ACh signal by the FT-ICR-MS analysis even after normalisation with the internal standard. This ACh detection was seemed to be induced by the instantaneous and inevitable leaking of the adjacent embedding medium containing extracellular ACh to the minute tissue clefts during the thaw-mounting or matrix spraying. The data of MALDI-IMS are often normalised by root mean square and total ion current method. However, these normalisation methods might not be suitable with ACh signal in this study because the ACh intensity of the FT-ICR-MS was quite small and was not predominant compared with the entire ion peaks [37]. Källback et al. [38] previously reported that utilisation of a deuterated internal standard yield the significantly better S/N ratio than usual normalisation in the MALDI-IMS analysis to visualise a drug distribution in animal lung tissues. To visualise ACh in a more micro-scale level in lung, the spatial resolution with good S/N in the ACh imaging can be improved by the better normalisation using deuterium-labelled matrix or deuterated ACh compound as an internal standard as reported by several previous studies [19, 20, 38]. In addition, this limitation could be overcome by using a model animal with larger lung components than a mouse, such as a rabbit or a canine. This imaging method is also unsuitable for the real-time analysis of lungs of living animals; therefore, we could not reveal dynamic changes in ACh after sensitisation by time-lapse measurements. Despite these limitations, this is a useful imaging technique and, although not utilised in this study, it has a unique advantage of being able to simultaneously visualise several molecules or compounds, including drugs [39], in the same tissue. Previous studies have reported successful visualisation of the distribution and quantity of administrated anti-asthma drugs such as tiotropium [40, 41] and corticosterone [42] in mouse lung tissues by IMS, while demonstrating that steroid medication suppressed ACh secretion induced by electrical stimuli of the vagus nerve [43]. Thus, in a future study, the simultaneous imaging of ACh and anti-asthma drugs by our FT-ICR-MS technique can investigate the effective administration method of drugs and assess ACh distribution changes in the lungs of asthma model mice during treatment with various therapeutic asthma drugs. We hope that the established imaging approach with FT-ICR-MS reported here highlights the significance of ACh in asthma pathogenesis and will contribute to further elucidating the pathophysiology of asthma.

## Electronic supplementary material

ESM 1 (PDF 1518 kb)
