# Supplementary material for: Imaging mass spectrometry to visualise increased acetylcholine in lungs of asthma model mice
Source: Anal Bioanal Chem. 2020 May 4;412(18):4327–41. doi: 10.1007/s00216-020-02670-0 (PMC7320054; doi:10.1007/s00216-020-02670-0)
Supplement: Supplementary file 1 — (PDF 1518 kb) [file 216_2020_2670_MOESM1_ESM.pdf]

Analytical and Bioanalytical Chemistry

Electronic Supplementary Material

Imaging mass spectrometry visualises increased acetylcholine in lungs of asthma model mice

Takeshi Matsuda, Yuzo Suzuki, Tomoyuki Fujisawa, Yasunori Suga, Nobuyuki Saito, Takafumi Suda, Ikuko Yao

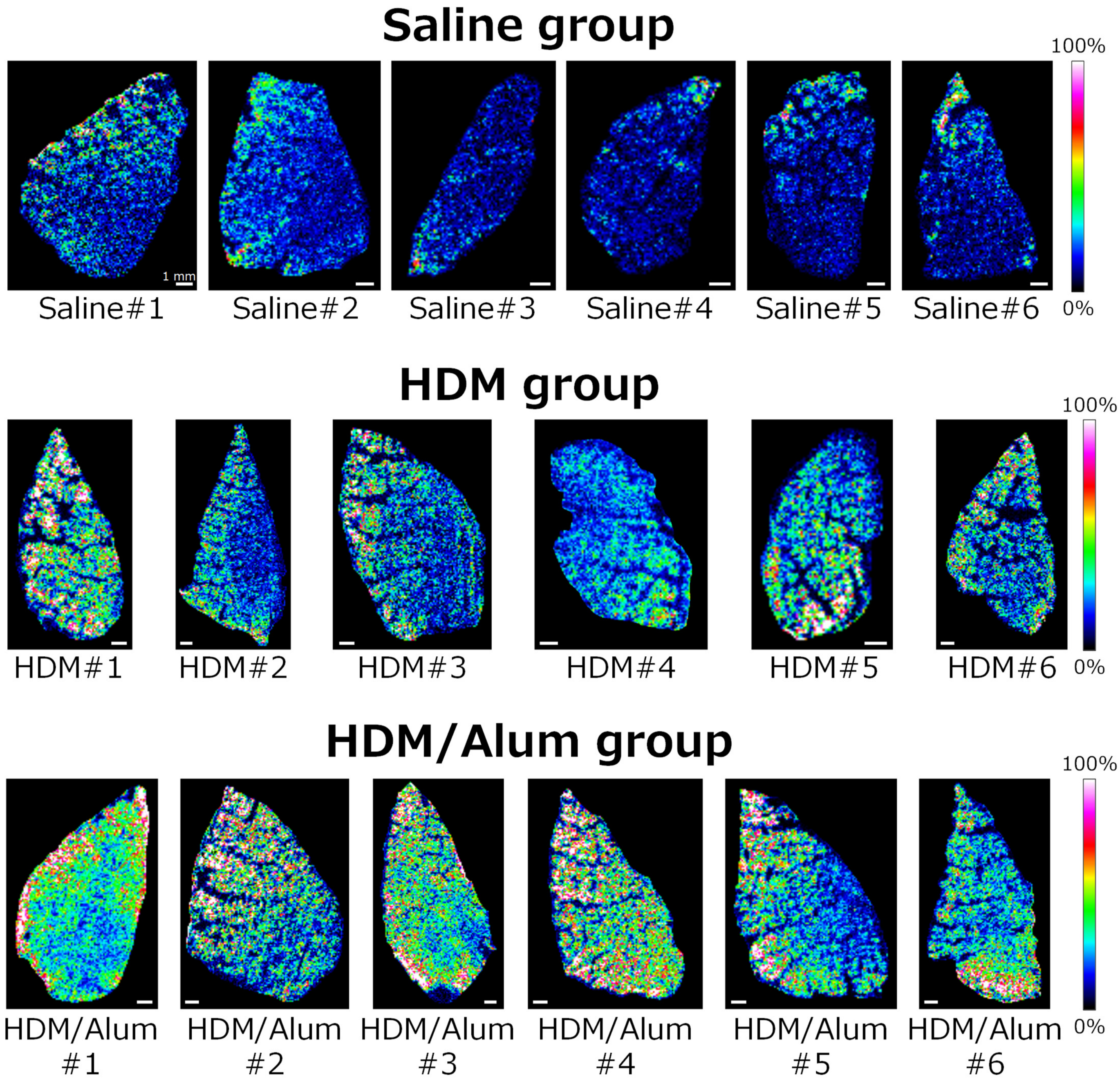

Fig. S1 ACh distribution patterns on lung tissues of asthma model mice

The representative ACh images on lung tissues from each mouse (N = 6 mice/group). ACh was visualised by the ACh imaging with FT-ICR-MS. ACh was distributed throughout the lung in a relatively uniform manner with some heterogeneity. The ACh visualisation revealed a clear tendency of the ACh elevation with the asthma severity, while there was modest individual difference between animals in an experimental group. The amount of ACh was increased in the HDM-sensitised mice compared with the saline-treated mice, and Alum adjuvant significantly promoted the ACh increase. Scale bars: 1 mm. HDM, house dust mite
